# Genetic relatedness, virulence, and drug susceptibility of *Campylobacter* isolated from water and wild birds

**DOI:** 10.3389/fcimb.2022.1005085

**Published:** 2022-11-25

**Authors:** Małgorzata Andrzejewska, Katarzyna Grudlewska-Buda, Dorota Śpica, Krzysztof Skowron, Małgorzata Ćwiklińska-Jurkowska, Małgorzata Szady-Grad, Piotr Indykiewicz, Natalia Wiktorczyk-Kapischke, Jacek J. Klawe

**Affiliations:** ^1^ Department of Hygiene, Epidemiology, Ergonomy and Postgraduate Education, Ludwik Rydygier Collegium Medicum in Bydgoszcz Nicolaus Copernicus University in Torun, Bydgoszcz, Poland; ^2^ Department of Microbiology, Ludwik Rydygier Collegium Medicum in Bydgoszcz Nicolaus Copernicus University in Torun, Bydgoszcz, Poland; ^3^ Department of Biostatistics and Theory of Biomedical Systems, Ludwik Rydygier Collegium Medicum in Bydgoszcz Nicolaus Copernicus University in Torun, Bydgoszcz, Poland; ^4^ Department of Biology and Animal Environment, Bydgoszcz University of Science and Technology, Bydgoszcz, Poland

**Keywords:** *Campylobacter*, water, birds, virulence genes, drug susceptibility, relatedness

## Abstract

**Introduction:**

This study aimed to identify the characteristics of Campylobacter isolated from wild birds (Black-headed gulls Chroicocephalus ridibundus and Great tits Parus major) and collect surface water samples (from rivers, ponds, ornamental lakes, freshwater beaches). Research material included 33 Campylobacter isolates. All the strains were isolated by different monitoring and surveillance plans.

**Methods:**

The prevalence of selected genes (flaA, cadF, iam, cdtB, wlaN, sodB, tet0) encoding virulence factors and resistance among Campylobacter spp. was assessed by the PCR method. The genetic similarities of isolates were determined by Pulsed-Field Gel Electrophoresis (PFGE). The susceptibility of Campylobacter isolates to clinically important antimicrobials: erythromycin, tetracycline, and ciprofloxacin, previously assessed by E-test, was presented in the form of drug susceptibility profiles depending on the origin of the isolates.

**Results:**

The cadF, flaA, cdtB, and sodB genes exhibited the highest detection rate. Statistically significant differences between the presence of wlaN virulence genes were noted among different species of the isolates. No genetically identical isolates were found. The most numerous antibiotic susceptibility profile included strains susceptible to all antibiotics studied (profile A-33.3%). The second most common were the tetracycline - and ciprofloxacin-resistant (profile B-27.2%), and tetracycline-resistant profile (C-24.2%) respectively.

**Discussion:**

The study revealed the virulent properties of Campylobacter isolated from water samples, and wild birds, and high resistance rates to tetracycline, and fluoroquinolones. The lack of genetic relatedness among strains isolated from water, and birds may indicate other sources of surface water contamination with Campylobacter bacteria than birds. The presence of Campylobacter spp. in wild birds could also have other environmental origins.

## Introduction

*Campylobacter* spp. is an important microorganism causing foodborne illnesses in developed and developing countries (Truccollo et al., 2001; Scallan et al., 2011; Raeisi et al., 2017). Campylobacteriosis is the most commonly reported gastrointestinal human infection in the EU since 2005. In 2019, the number of confirmed cases of human campylobacteriosis was 220,682 corresponding to the EU notification rate of 59.7 per 100,000 population (EFSA, 2019). In the United States *Campylobacter* spp. is responsible for approximately 1 million diarrheal illnesses annually. Therefore the zoonoses are of economic and public health concern (Scallan et al., 2011). Human campylobacteriosis is predominantly caused by *Campylobacter jejuni* (*C. jejuni*), and *Campylobacter coli* (*C. coli*), although *C. jejuni* is responsible for the majority of these infections (EFSA, 2019).

*Campylobacter* spp. is commensally widespread in the intestines of wild and domesticated animals, resulting in contamination of the environment, including water sources (Mughini Gras et al., 2012). The most common route of human transmission is foodborne exposure. Transmission to people may occur through the consumption of undercooked meat, especially chicken, or unpasteurized milk (Abebe et al., 2020).

One of the media of *Campylobacter* spp. that is responsible for the dissemination of this bacteria in the environment is surface water, on which bacteria can be found along with wastewater, the feces of wild birds, and waste from factory farms (Whiley et al., 2013). Drinking water contaminated with *Campylobacter* spp., or untreated water ingested from unknown sources is often the source of outbreaks in humans (Hyllestad et al., 2020; Gilpin et al., 2020).

An important reservoir of bacteria of the genus *Campylobacter* is the digestive tract of birds, whose body temperature (42°C) creates excellent conditions for their development (Mohan, 2015). Free-living birds, especially migratory species, can become long-distance vectors for *Campylobacter* spp. strains (also resistant to antibiotics), which can be transmitted to humans and livestock. Due to high mobility, birds can contaminate pastures, cultivated fields, and surface waters with their feces (Mohan, 2015; Zeballos-Gross et al., 2021). A great variety of animal and environmental reservoirs of *Campylobacter* spp. makes the epidemiology caused by this bacteria extremely complex.

Most current studies on *Campylobacter* draw attention to poultry meat as the most important carrier of *Campylobacter* bacteria. However, less attention is paid to the phenotypic and molecular characteristics of strains from other potential sources such as surface water or wild animal including birds. The lack of genetic information on *Campylobacter* spp. of wild birds and their aquatic origin in Poland highlights the need to monitor their genotypes and potential relationships. Likewise, the surveillance of drug-resistant *Campylobacter* isolated from the environment is of paramount importance due to the potential health risks for people using surface water for recreational purposes and the possibility of the long-distance spread of pathogens by migratory birds. Therefore, the study aimed to evaluate the prevalence of selected gene-encoding virulence factors among *Campylobacter* isolated from water sources and wild birds. Additionally, drug susceptibility profiles and genetic similarity were evaluated.

## Material and methods

### Material

A total of 185 surface water samples were obtained in Northern Poland from 2017 to 2020. Water samples were collected from rivers, ponds, ornamental lakes, and freshwater beaches in spring, summer, and early autumn. The samples were purchased from different types of surface water. The sites were also selected based on their geographical location. The number of samples collected during each year of the study was as follows: October 2017 - 20 samples, March, July, and October 2018 - 63 samples, March, July, and October 2019 - 63 samples, and March and July 2020 - 39 water samples.

*Campylobacter* isolates from water sources were compared to previously collected isolates obtained from two species of wild migratory birds (Black-headed gulls *Chroicocephalus ridibundus* and Great tits *Parus major*). Only isolates detected in the study area were taken into consideration. Detailed information on the detection of *Campylobacter* in selected bird species has been described previously in other published studies (Tryjanowski et al., 2020; Indykiewicz et al., 2021). Altogether, 33 *Campylobacter* isolates were selected for further investigation.

### Isolation of Campylobacter from water

Isolation of *Campylobacter* spp. from water samples was performed according to International Organization for Standardisation (2019). We filtered 100 ml of water sample through 0.45μm filters (Merc Millipore, Burlington, USA) and then each filter was transferred to 90 ml of Bolton broth (Oxoid Limited, Basingstoke, United Kingdom). The broth was incubated at 41°C in a microaerophilic atmosphere (Generbox microaer-BioMerieux, Marcy l’Etoile, France) for 48 hours and after preincubation 10μl of culture was performed on a solid CCDA medium (Oxoid Limited, Basingstoke, United Kingdom). Characteristic growth from the CCDA plates was transferred to a blood plate (Columbia agar containing 5% cattle blood, Oxoid Limited, Basingstoke, United Kingdom) and incubated overnight at 41°C. Colonies suspected as *Campylobacter* spp. were examined for cell morphology, motility, and oxidase reactions. Putative *Campylobacter* colonies were frozen at -80 in Microbanks (Pro-Lab Diagnostics, Birkenhead, United Kingdom) until species differentiation.

### Species identification

The identification of isolates was performed with a Microflex LT/SH mass spectrometer (Bruker, Billerica, USA) using the MALDI Biotyper software package (version 4.1). The reference Bruker Taxonomy database (Bruker, Billerica, USA) and default parameter settings followed previous methods by Schulthess et al. (2013). The Bruker bacterial test standard (BTS; Bruker, Billerica, USA) was used for validation according to the manufacturer’s instructions.

### Genetic similarity

The genetic similarity between the selected isolates of *Campylobacter* spp. was determined with Pulsed-Field Gel Electrophoresis (PFGE). The procedure for genotyping was performed in accordance with the Standard Operating Procedure for PulseNet PFGE *Campylobacter jejuni* with the recommended *smaI* enzyme (McDougal et al., 2003; Centers for Disease Control and Prevention (CDC), 2017). Based on the obtained results Jaccard similarities were calculated. Clustering was obtained by the application of agglomerative hierarchical cluster analysis on a set of distances (1-similarities) by the weighted pair group method with arithmetic mean. Procedure hclust from the R package was used for clustering and plotting the dendrogram.

### DNA isolation

To carry out DNA isolation, the following actions were performed: several bacterial colonies from the mCCDA were placed in 100μl of NaCl solution, then 45μl of chelating resin solution Chelex -100 (BioRad, Hercules, USA) with a concentration of 20% was added. The mixture was placed in a thermoblock at 100°C and heated for 10 minutes. After heating, the mixture was quickly cooled by inserting a sample into the ice. The sample was centrifuged for 10 minutes (RPM = 13000). The supernatant was collected and stored at -20°C (de Lamballerie et al., 1992).

### Detection of genes responsible for virulence and resistance

Genotypic characterization of strains has been carried out based on an assessment of the presence of the *flaA*, *cadF*, *cdtB*, *iam*, *wlaN*, and *sodB* genes responsible for coding virulence factors such as cell motility, adherence to the intestinal epithelium, invasiveness, toxin production, molecular mimicry, response to oxidative stress and *tet0* gene associated with tetracycline resistance. The PCR technique and starter kits described in earlier literature were used for this purpose (Nachamkin et al., 1993; Konkel et al., 1999; Linton et al., 2000; Bang et al., 2001; Carvalho et al., 2001; Datta et al., 2003; Biswas et al., 2011; Obeng et al., 2012, **Table 1**).

**Table 1:** Sequences of primers, annealing temperatures, and product sizes.

Primers	Sequence (5’ → 3’)	Product (bp)	Annealing temperature	References
*cadF*-F *cadF*-R	TGGAGGGTAATTTAGATATG CTAATACCTAAAGTTGAAAC	400	45˚C	(Konkel et al., 1999)
*flaA*-F *flaA*-R	GGATTTCGTATTAACACAAATGGTGC CTGTAGTAATCTTAAAACATTTTG	1728	55˚C	(Nachamkin et al., 1993)
*cdtB*-F *cdtB*-R	GTTAAAATCCCCTGCTATCAACCA GTTGGCACTTGGAATTTGCAAGGC	495	42˚C	(Bang et al., 2001)
*iam*-F *iam*-R	GCGCAAAATATTATCACCC TTCACGACTACTATGCGG	518	52˚C	(Carvalho et al., 2001)
*wlaN*-F *wlaN*-R	TTAAGAGCAAGATATGAAGGTG CCATTTGAATTGATATTTTTG	672	46˚C	(Linton et al., 2000; Datta et al., 2003)
*sodB*-F *sodB*-R	ATGATACCAATGCTTTTGGTGATTT TAATACGACTCACTATAGGGCATTTGCATAAAAGCTAACTGATCC	638	50˚C	(Biswas et al., 2011)
*tet0*-F *tet0*-R	GCGTTTTGTTTATGTGCG ATGGACAACCCGACAGAAG	559	54°C	(Obeng et al., 2012)

The reaction mixture of each PCR consisted of: -5μl DreamTaq PCR Buffer (Thermo Fisher Scientific, Waltham, Massachusetts, US) - 0,5μl dNTPs (10mM Thermo Fisher Scientific, Waltham, Massachusetts, US), -1μl of each starter (10μM - DNA Sequencing and Synthesis Laboratory IBB PAN Oligonucleotide, Warsaw, Poland) - 0,5μm (1U) Dream Taq DNA polymerase (Thermo Fisher Scientific, Waltham, Massachusetts, US), - 2μl of DNA tested, - 15μl nuclease free water. The final volume of the reaction mixture was 25μl. The thermal profile of each amplification reaction was based on publications (Nachamkin et al., 1993; Konkel et al., 1999; Linton et al., 2000; Bang et al., 2001; Carvalho et al., 2001; Datta et al., 2003; Biswas et al., 2011; Obeng et al., 2012, **Table 1**). The visualization of PCR reaction products was carried out using electrophoresis in 1.5% agarose gel with Midori Green DNA Stain (Nippon Genetics, Duren, Germany). The size of the DNA product was compared with the DNA size marker 100bp (Thermo Fisher Scientific, Waltham, MA, US).

### Drug susceptibility

Both types of material (water and birds *Campylobacter* isolates) were tested for antimicrobial susceptibility using E-test (BioMerieux, Marcy l’Etoile, France) on Mueller-Hinton agar with 5% defibrinated horse blood (Oxoid Limited, Basingstoke, UK). The incubation of Mueller-Hinton plates was undertaken in accordance with the manufacturer’s recommendations. The susceptibility of *Campylobacter* water isolates to clinically important antimicrobials: erythromycin, tetracycline, and ciprofloxacin were compared with the previous result obtained from *Campylobacter* birds isolates and presented in the form of drug susceptibility profiles depending on the origin of the isolates. MIC values were determined according to the Clinical and Laboratory Standard Institute’s guidelines (CLSI, 2015). The thresholds for *Campylobacter* resistance were set as follows: erythromycin 32 µg/mL, tetracycline 16 µg/mL, and ciprofloxacin 4 µg/mL.

### Statistical methods

Statistical analysis was performed using the Statistica 13.3 program (TIBCO Software Inc. (2017). Statistica (data analysis software system), version 13. http://statistica.io). The Chi-square test and the exact Fisher test for small sample sizes were applied to examine the differences in the proportions of virulence genes between species (*C. jejuni*, *C. coli* and *C. lari*), and between wild birds and water *Campylobacter* isolates. A significance level of p = 0.05 was taken.

## Results

### Prevalence of Campylobacter spp. in water samples

The results of the prevalence tests of *Campylobacter* isolated from surface water in Poland from 2017 to 2020 indicated the presence of examined bacteria in 15 (8.1%) of the samples. MALDI-TOF Mass Spectrometry, which was used to detect and differentiate *Campylobacter*, confirmed the presence of two species: *C. jejuni* and *C. coli*. The frequency of *C. jejuni* in the examined samples was 73%, *C. coli* was found in 27% of the analyzed samples. The highest prevalence of *Campylobacter* spp. (12.5%) was noted in river samples followed by ponds (10.5%) and beaches (4.1%). The data are presented in **Table 2**.

**Table 2:** Prevalence of *Campylobacter* isolated from surface water samples in Poland between 2017-2020.

Sample type	No. of samples tested	No. (%) of samples positive for *Campylobacter*	No. (%) of samples positive for *C. jejuni*	No. (%) of samples positive for *C. coli*
Rivers	8	1 (12.5)	1 (100)	0
Ponds and ornamental lakes	105	11 (10.5)	8 (73)	3 (27.3)
Freshwater beaches	72	3 (4.1)	2 (67)	1 (33)
Total	185	15 (8.1)	11 (73)	4 (27)

### Evaluation of genetic (PFGE) similarity levels

**Figure 1:**
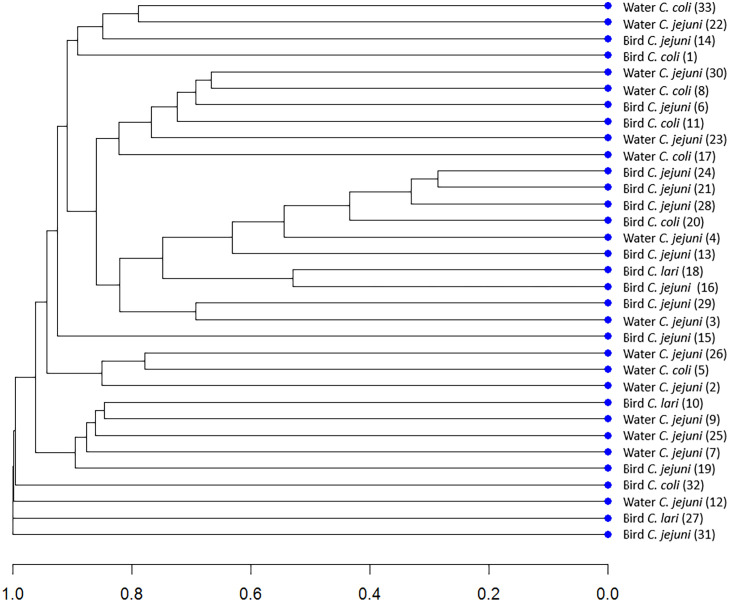
Dendrogram determining the degree of genetic dissimilarity of the tested isolates of *Campylobacter* spp. from water and wild birds.

The analysis of the genetic similarity by the PFGE method allowed the creation of 33 characteristic band patterns for the studied isolates. **Figure 1** shows a dendrogram determining the degree of genetic similarity of the tested isolates of *Campylobacter* spp. obtained from water and wild birds. Defining similarity with the PFGE method using a cut-off point of 0.8 is considered a gold standard (McDougal et al., 2003). Due to this fact, we used this cut-off point value for PFGE results. No genetically identical isolates were found. The 33 strains formed unique band patterns and were not classified into any of the clusters, with the assumed cut-off level.

### Frequency of virulence and resistance genes

The distribution of virulence genes in *Campylobacter* spp. isolated from environmental sources is presented in **Table 3**. In general, the most common markers, present in all isolates, were *cadF*, *flaA* genes, which are associated with adhesion and motility. In addition, the *sodB* gene was present in all *Campylobacter* isolates taken from water samples. High levels of the *Campylobacter cdtB* virulence marker were also noted (100% in *C. coli* isolates both from water and birds). Lower levels of the pathogenic genes *tet0* (54.5%) and *iam* (51.5%) were noted in *Campylobacter* isolates obtained for this study. The most diverse virulence gene in terms of occurrence was the *wlaN* gene, its detection rate varied from 0 among *C. lari* strains isolated from birds to 100% among water-derived *C. coli* isolates. Considering the wild birds’ isolates, the significant difference between *C. jejuni* vs *C. coli* and between *C. jejuni* vs *C. lari* for *wlaN* gene (associated with molecular mimicry) was found. However, having examined water isolates and all considered genes, no significant difference between any pair of species was found. Additionally, no statistically significant differences between the presence of virulence markers among isolates from water and birds have been identified (**Table 3**).

**Table 3:** Distribution of virulence genes in *Campylobacter* spp. isolated from environmental sources.

Origin	No. of Isolates	*Campylobacter* Virulence Factor No. of Isolates with The Occurrence of The Gene (%)						
		*flaA*	*cadF*	*iam*	*cdtB*	wlaN*	*sodB*	*tet0*
Birds	Total - 18	18 (100)	18 (100)	10 (55.5)	16 (88.8)	11 (61.1)	16 (88.9)	11 (61.1)
	*C. jejuni -* 11	11 (100)	11 (100)	7 (63.6)	10 (91)	10 (91)	9 (81.8)	7 (63.6)
	*C. coli -* 4	4 (100)	4 (100)	2 (50)	4 (100)	1 (25)	4 (100)	3 (75)
	*C. lari -* 3	3 (100)	3 (100)	1 (33.3)	2 (66.6)	0	3 (100)	1 (33.3)
Water	Total - 15	15 (100)	15 (100)	7 (46.6)	12 (80)	10 (66.6)	15 (100)	7 (46.7)
	*C. jejuni -* 11	11 (100)	11 (100)	4 (36.4)	8 (72.7)	6 (54.5)	11 (100)	5 (45.5)
	*C. coli -* 4	4 (100)	4 (100)	3 (75)	4 (100)	4 (100)	3 (100)	2 (50)
Total	33	33 (100)	33 (100)	17 (51.5)	28 (84.8)	21 (63.6)	31 (94)	18 (54.5)

*Statistically significant differences between the presence of virulence marker *wlaN* among different species have been identified for wild bird isolates: *C. jejuni/C. coli* (p = 0.033) and *C. jejuni/C. lari* (p = 0.011).

### Drug susceptibility

The results of antimicrobial resistance in relation to sample origin are shown in **Table 4**. The most numerous antibiotic susceptibility profile included strains susceptible to all antibiotics studied (profile A-33.3%). The second most common was the tetracycline- and ciprofloxacin-resistant (profile B-27.2%). In our study tetracycline-resistant profile was also observed with high frequency (C-24.2%) respectively. All the isolates that were phenotypically resistant to tetracycline harbored the *tetO* gene. Resistance to ciprofloxacin and susceptibility to other examine antibiotics was less common (profile D-9.1%). One strain of *C. coli* obtained from water presented simultaneous resistance to ciprofloxacin and erythromycin. One C. *jejuni* isolated from a bird presented simultaneous resistance to ciprofloxacin, tetracycline, and erythromycin. No significant differences between the proportions of water and wild birds were found in terms of drug susceptibility profiles (**Table 4**).

**Table 4:** Drug susceptibility profiles of the tested *Campylobacter* spp. isolated from various sources.

Profile	Drug susceptibility	No. of isolates			Total N = 33(%)
		Water N = 15 (%)	Wild birds N = 18 (%)	p	
A	S*: TET, CIP, ERY, R*: —	5 (33.3)	6 (33.3)	0.7411	11 (33.3)
B	S: ERY R: TET, CIP	5 (33.3)	4 (22.2)	0.2405	9 (27.2)
C	S: ERY, CIP R: TET	3 (20)	5 (27.8)	0.4587	8 (24.2)
D	S: ERY, TET R: CIP	1 (6.7)	2 (11.1)	0.5702	3 (9.1)
E	S: TET R: ERY, CIP	1 (6.7)	0	0.4545	1 (3)
F	S: — R: ERY, CIP, TET	0	1 (6.7)	0.5455	1 (3)

S, susceptible isolates; R, resistant isolates; TET, tetracycline; CIP, ciprofloxacin; ERY, erythromycin.

## Discussion

In this research, a comparative study was undertaken, examining *Campylobacter* isolates from two different sources: surface waters and wild birds from Northern Poland.

In total, 15 out of 185 (8.1%) water samples were positive for *Campylobacter* spp., and the occurrence of the examined bacteria in the surface water was lower than reported previously by Szczepańska et al. in the same study area in Poland (Szczepańska et al., 2017). Nevertheless, river contamination was the highest in both studies. In the present study, *Campylobacter* was detected in 3 out of 72 freshwater beach samples (4.1%), which may increase the risk of outbreaks during swimming. An even higher *Campylobacter* detection rate was observed in studies by Guy et al. at the lake beaches used for water recreation in Canada (Guy et al., 2018). The proportion of positive water samples was estimated to be 33.9% for *C. jejuni* in that study. *Campylobacter* detection in surface waters research varies depending on the season of sampling and the source. *Campylobacter* prevalence was shown to be high in agricultural waters (77%), and in recreational waters (46%) in studies by Mulder et al., 2020 in the Netherlands (Mulder et al., 2020).

This is the first report from the study area on the application of PFGE for genotyping of *Campylobacter* isolated from water and animal reservoirs. PFGE is currently considered a gold standard method that has proven to be a useful epidemiological tool for the genetic discrimination of bacterial strains (Noormohamed and Fakhr, 2014; Tang et al., 2019). In the available literature, there are no reports investigating the genetic similarity of *Campylobacter* strains isolated from wild birds and water sources, with the PFGE method. The isolates analyzed in our study were not genetically similar. The lack of genetic relatedness among strains isolated from water and birds may indicate other sources of surface water contamination with *Campylobacter* bacteria than birds. The presence of *Campylobacter* spp. in wild birds could have other environmental origins. More investigation would provide data on the epidemiology of *Campylobacter* in the study area.

In previous studies by Mulder et al., a multilocus sequence typing (MLST) scheme was developed to characterize isolates from water sources in the Netherlands (Mulder et al., 2020). Tracking the strains according to their origin showed that water isolates were attributed mainly to wild birds and poultry. Wild birds’ contribution was especially high among isolates from recreational waters, which is in contradiction with our results, which showed no similarity between the freshwater beaches and birds. A high genetic diversity of strains isolated from different species of birds in China was observed in studies by Du et al. (2019). *C. jejuni* isolates from wild birds in this study were tested by MLST. The majority of the STs in this study did not belong to any clonal complex. Moreover, a great genetic diversity between isolated strains was observed (Iglesias-Torrens et al., 2018) in a comparative study among *C. jejuni* strains from humans, broilers, and wild birds from Catalonia (performed using the PFGE method).

Data on the occurrence of virulence and resistance markers in *Campylobacter* isolates are mainly related to the detection of genes in *Campylobacter* poultry or human samples. The number of studies that examine the virulent properties of environmental *Campylobacter* stains is limited. Overall, the most common virulence genes in this study were *cadF, flaA*, and *cdtB*. The protein encoded by the *cadF* gene is responsible for binding to the fibronectin of intestinal epithelial enterocytes, enabling the process of internalization of the bacterial cell. The ability to move is determined by the presence of cilia, composed of flagellin encoded by the *flaA* gene. The component of cdt responsible for the toxic effect is the *cdtB* gene, whose product initiates a cascade leading to cell cycle inhibition (Nachamkin et al., 1993; Konkel et al., 1999; Bang et al., 2001). Our results reflect a report from Egypt, which also identified *flaA* and *cdtB* genes in 100% of *C. jejuni* isolated from pigeons (Abd El-Hamid et al., 2019). Research conducted by Iglesias-Torrens et al. showed that the *cdtB* gene (involved in toxin production) was detected in 92% of *C. jejuni* isolates from wild birds in Spain (Iglesias-Torrens et al., 2018). A high level of detection of *cadF* gene was also observed. Similarly, in the study of Shyaka et al. investigating the virulence-associated factors in *Campylobacter* from wild birds detection of *cadF*, *flaA*, and *ctdB* genes was confirmed in all *C. jejuni* obtained from pigeons and crows (Shyaka et al., 2015). The opposite data were presented in virulence studies of strains derived from rivers in Africa, where detection of *cadF*, *flaA*, and *cdtB* markers was very low (Oh et al., 2019).

In our study, the *iam* gene represented the virulence marker responsible for the invasion of the host cell. The *iam* gene was detected in *Campylobacter* isolates recovered from birds and water with 55.5 and 46.6% frequency respectively. *Campylobacter* isolate detection rate was higher in *C. coli* isolates in water. The same result was described in a study by Igwaran and Okoh (2020).

*Campylobacter* spp. are microaerophilic stress-sensitive pathogens that may cause a significant number of cases of human *gastroenteritis* worldwide (Oh et al., 2019). The gene involved in the stress response is *sodB.* Many authors emphasize the role of the *sodB* gene and proteins produced by this gene in the initial colonization of poultry intestines and overcoming stress conditions during the passage through the gastrointestinal tract in humans. *SodB* mutants show increased sensitivity to oxidative stress. This is confirmed by studies in which a high percentage of the presence of the *sodB* gene was detected among isolates from poultry carcasses or humans (Wieczorek et al., 2018; Oh et al., 2019).

*WlaN* was selected as a pathogenic gene responsible for the ganglioside mimicking Guillain-Barré syndrome. A limited number of studies have reported *wlaN* gene detection in *Campylobacte*r obtained from environmental sources. Our results showed that this marker was present in 61.1% of the tested *Campylobacter* isolates from birds and in 66.6% of isolates obtained from water samples. According to the investigation by Gargiulo et al., this gene was present in only 17.5% of *Campylobacter* isolates from common teals (Gargiulo et al., 2011). The detection of *wlaN* markers was not confirmed in *C. coli* isolated from those migratory birds. Similarly, the lower prevalence of the *wlaN* gene (11.3%) was demonstrated in *C. jejuni* isolates obtained from wild birds in South Korea. The lack of prevalence of the *wlaN* gene in *C. coli* stains in the same study is noteworthy (Wei et al., 2019). In the study by Guirado et al., the percentage of *Campylobacter* strains in which *wlaN* was detected was also low (6%) (Guirado et al., 2020). The studies mentioned above are inconsistent with our observations and require further research. Despite the high incidence of the *wlaN* gene among *C. jejuni* and *C. coli* strains from both studied environments, this gene was not detected among *C. lari* strains.

The *tetO* gene, which is responsible for the build-up of resistance, is located in the plasmid or chromosomal DNA of bacteria. The product of this gene, the TetO protein, causes a decrease in the binding capacity of tetracyclines to the 30S subunit of the ribosome, resulting in the lack of inhibition of polypeptide chain synthesis in the bacterial cell. In the current study, 61.1% of the birds and 46.7% of the water *Campylobacter* isolates harbored the *tetO* gene. The findings of our study corroborate a report by Chukwu et al. who observed a *tetO* resistance marker in 40% of *Campylobacter* from water samples in South Africa (Chukwu et al., 2019).

The increasing resistance of *Campylobacter* spp. has become a serious problem, especially with antibiotics used during standard therapy of human campylobacteriosis. According to EFSA, the data obtained from *C. jejuni* and *C. coli* from human and animal origins between 2018 and 2019, showed very high levels of resistance to fluoroquinolones and tetracycline. What is more, combined resistance to ciprofloxacin and erythromycin in animal *Campylobacter* isolates raises concerns (EFSA, 2021). The *Campylobacter* spp. examined in our study were obtained from different sources but had similar antibiotic resistance profiles, despite the demonstrated lack of genetic similarity between them. A comparable pattern was observed in the study by Olkola et al. where the percentage of antibiotic-resistant *Campylobacter* isolates was similar at 6.3% (natural waters), and 11.4% (wild birds), respectively, with no apparent genetic similarity assessed by MLST.

In general, most isolates in our study (21 out of 33 - 63.6%) exhibited profiles associated with resistance to one or more antibiotics. The research confirmed a high level of tetracycline and ciprofloxacin resistance among *Campylobacter* environmental isolates. Similar results were obtained previously in Poland, resistance profile (ciprofloxacin + tetracycline) in *C. jejuni* and *C. coli* from surface water was estimated at 30 and 16.7% respectively (Szczepańska et al., 2017). In the studies by Olkola et al. tetracycline resistance was most commonly observed in the wild birds and water samples, while resistance to erythromycin was not detected in *C. jejuni* environmental isolates (Olkkola et al., 2016). In our study, the single strains derived from water and birds were resistant to erythromycin (an antibiotic crucial in campylobacteriosis treatment), as well as to other examined antibiotics. Multidrug resistance of *Campylobacter* species isolated from the environment has been described previously and these studies raised concerns (Iglesias-Torrens et al., 2018; Igwaran and Okoh, 2020).

This is the first study examining the occurrence of genes responsible for virulence and resistance at various stages of pathogenesis among *Campylobacter* isolated from birds and the aquatic environment in Poland. Our findings reveal that the number of *Campylobacter* isolates recovered from water samples and birds with key pathogenic factors responsible for motility, adherence, toxin production, invasiveness, molecular mimicry, and resistance to antibiotics was significant. However, differences in the gene frequency between different species of *Campylobacter* have also been described, which needs to be further verified. The existence of *Campylobacter* species containing genes associated with virulence in water (especially bathing beaches) was proven to be a significant potential source of human campylobacteriosis.

This study investigated the possible transmission of the important human pathogen between two environments: water and wild birds. Based on molecular subtyping we concluded that the exchange of this pathogen between wildlife and water sources is infrequent. Migratory birds may spread virulent and resistant *Campylobacter* spp. to the environment but due to the lack of similarity between isolates, other sources of water contamination should be taken into consideration. A greater number of isolates and sources should be investigated in prospective future studies to explain the circulation of this pathogen in the environment.

## Data availability statement

The raw data supporting the conclusions of this article will be made available by the authors, without undue reservation.

## Author contributions

Conceptualization, MA, KG-B and KS, writing - original draft preparation, MA and KG-B. Writing - review and editing, MA, KG-B, KS and MĆ-J, Methodology, MA, KG-B and KS, Investigation, MA, DŚ, KG-B, MĆ-J and NW-K. Sampling, DŚ, PI and MA. Statistics, MS-G. and MĆ-J. Supervision, JK. All authors contributed to the article and approved the submitted version.

## Funding

This research received funding from the “Excellence Initiative Research University” program at Nicolaus Copernicus University in Toruń.

## Conflict of interest

The authors declare that the research was conducted in the absence of any commercial or financial relationships that could be construed as a potential conflict of interest.

## Publisher’s note

All claims expressed in this article are solely those of the authors and do not necessarily represent those of their affiliated organizations, or those of the publisher, the editors and the reviewers. Any product that may be evaluated in this article, or claim that may be made by its manufacturer, is not guaranteed or endorsed by the publisher.
